# Effect and Nursing Satisfaction of Bedside Nursing Combined with Detail Nursing in Clinical Nursing of Gastroenterology Department

**DOI:** 10.1155/2021/6839555

**Published:** 2021-05-31

**Authors:** Hongyan Ai, Shuqin Peng

**Affiliations:** ^1^Department of Gastroenterology, Linyi Central Hospital, Linyi 276400, Shandong Province, China; ^2^Department of Traditional Chinese Medicine, Second Hospital of Liaocheng, Linqing, Liaocheng 252600, Shandong Province, China

## Abstract

**Objective:**

The purpose of the study was to investigate the therapeutic effect and nursing satisfaction of bedside nursing combined with detail nursing in the gastroenterology department.

**Methods:**

112 patients with gastrointestinal diseases admitted to our hospital from November 2018 to November 2019 were selected as the study subjects and randomly divided into a research group (*n* = 56) and reference group (*n* = 56). The reference group received routine clinical nursing, while on this basis, the research group received bedside nursing combined with detail nursing. After that, the clinical nursing effects of the two groups were compared.

**Results:**

There were no significant differences in sex ratio, age, BMI, smoking history, drinking history, marital status, and disease types between the two groups (*P* > 0.05). The VAS scores in the two groups after intervention were significantly lower than those before intervention (*P* < 0.01), and the VAS scores in the research group after intervention were significantly lower than those in the reference group (*P* < 0.01). The nursing ability, nursing skills, and nursing responsibility in the research group were significantly higher than those in the reference group (*P* < 0.01). There were no significant differences between the two groups in the number of patients who were satisfied and needed improvement (*P* > 0.05). Besides, the number of very satisfied cases in the research group was significantly higher than that in the reference group (*P* < 0.05), and the number of unsatisfied cases was significantly lower than that in the reference group (*P* < 0.05). The total incidence of clinical adverse events in the research group was significantly lower than that in the reference group (*P* < 0.01). The gastrointestinal diseases related knowledge scores after intervention were significantly higher than those before intervention (*P* < 0.01), and the gastrointestinal diseases related knowledge scores after intervention in the research group were significantly higher than those in the reference group (*P* < 0.01). The GQOLI-74 scores after intervention in the two groups were significantly higher than those before intervention (*P* < 0.01), and the GQOLI-74 scores after intervention in the research group were significantly higher than those in the reference group (*P* < 0.01).

**Conclusion:**

The application of bedside nursing mode combined with detail nursing in gastrointestinal diseases can effectively reduce patients' pains, as well as the incidence of clinical adverse events, and improve patients' life quality, with definite curative effect, which is worthy of promotion and application.

## 1. Introduction

With the accelerating rhythm of people's life, the number of patients with digestive diseases has been increasing. Gastroenterology refers to the three-level clinical disciplines with stomach, esophagus, large and small intestines, gallbladder, and other diseases as the main contents, covering a wide range of diseases, and it has complex and sophisticated clinical treatment and nursing operation [1–3]. At present, patients not only put forward higher requirements for clinical treatment effect but also have higher requirements for daily nursing care. Due to the characteristics of high incidence and wide coverage, gastroenterology commonly occurs in all age groups; therefore, with improved treatment effect, the quality and level of clinical nursing care cannot be ignored [4–6]. Detail nursing complies with this requirement, and its measures can effectively improve patients' negative moods and make patients satisfied with the high-quality nursing services in hospital. Detail nursing is the inheritance and innovation based on the traditional clinical nursing, and it has been recognized by the society through optimizing the nursing process, changing the traditional doctor-patient communication mode, paying more attention to clinical details, improving the satisfaction of clinical nursing, creating a good nurse-patient relationship, and improving the overall treatment level of the hospital [7–9]. Bedside nursing is a brand-new nursing method that establishes responsibility system groups and carries out level-to-level administration, which can improve the responsibility consciousness of nursing staff to a certain extent, reduce the incidence of clinical adverse events, provide guarantee for clinical treatment, make hospital nursing services closer to patients, and establish a harmonious nurse-patient relationship. Based on this, this study further explores the clinical effect of bedside nursing combined with detail nursing in digestive diseases, and now the summary reports are as follows.

## 2. Materials and Methods

### 2.1. General Information

This study was approved by the Hospital Ethics Committee. 112 patients with gastrointestinal diseases admitted to our hospital from November 2018 to November 2019 were selected as the study subjects and randomly divided into a research group (*n* = 56) and control group (*n* = 56).

### 2.2. Inclusion Criteria

① Patients met the diagnostic criteria of digestive diseases. ② Patients had complete clinical data. ③ The patients and their families were informed of the purpose and process of this study and signed the informed consent.

### 2.3. Exclusion Criteria

① Patients had other organic lesions in the brain, heart, kidney, and liver. ② Patients had cognitive or communication disorders such as mental disorders. ③ Patients refused to cooperate with the study.

### 2.4. Methods

The clinical nursing was carried out in the reference group through advising patients to take medicine on time, keeping the ward environment clean and tidy, implementing dietary intervention for patients, and monitoring various vital signs.

The research group received bedside nursing combined with detail nursing on the basis of routine clinical nursing. Bedside nursing: ① primary nursing groups were established, whose members were composed of a head nurse and 3 or 4 nurses. The head nurse, serving as the group leader, arranged specific work according to the working ability of each nurse. Besides, the primary nurses performed comprehensive nursing in clinical propaganda and education, psychological counseling, medication guidance, and dietary intervention. ② Three-level quality control was adopted. The first level referred to that nurses timely found existing problems through self-inspection, self-evaluation, and other ways and then took effective measures to solve them. The second level referred to that the team leader timely checked each team member's nursing record sheets, patients' medication records, snd so forth, to supervise and correct their work. The third level referred to that department leaders developed specific work assessment mechanisms and guided the daily work of each nurse. ③ Departments equipped each group with a treatment vehicle and with the drugs and instruments needed in the treatment process. Detail nursing: ① medical staff should actively communicate with patients, eliminate their negative emotions, and explain the relevant precautions during hospitalization. In addition, medical staff should act gently with smiling when nursing patients so as to establish a good doctor-patient relationship and make them feel more love and care. ② Medical staff should pay attention to appearance, face patients with a positive and enthusiastic attitude, and leave a good mark for patients. ③ Medical staff should pay attention to their own language expression and tone of voice in clinical nursing to make patients more acceptable.

### 2.5. Observation Indexes

The clinical data of the two groups were compared and analyzed, which included gender, age, body mass index (BMI), smoking history, drinking history, marital status, and disease types.

The pain degree before and after intervention was evaluated by referring to the visual analogue scale (VAS) [10], with the total score of 10 points, and higher scores indicated higher pain degree.

The Clinical Nursing Quality Scale made by the department was adopted to evaluate the clinical nursing quality of the two groups, which included three items, such as nursing ability, nursing skills, and nursing responsibility, with each item totally scoring 50 points, and higher scores indicated better nursing quality.

The Patient Clinical Satisfaction Questionnaire prepared by the hospital was used, and the patients were guided to fill in it truthfully. According to the satisfaction level of clinical nursing, the questionnaire can be classified as being very satisfied, satisfied, needing improvement, and unsatisfied.

The incidence of clinical adverse events during hospitalization was statistically compared between the two groups.

The Patient Disease Related Knowledge Scale prepared by the department was adopted to evaluate the knowledge mastery of gastrointestinal diseases in the two groups before and after intervention, with the total score of 100 points, and higher scores indicated better patients' knowledge mastery of gastrointestinal diseases.

Referring to Generic Quality of Life Inventory-74 [11] (GQOLI-74), the life quality of the patients in the two groups before and after intervention was evaluated. The scale was composed of four scoring factors, such as psychological function, physical function, social function, and material life state, with the total score of 100 points, and higher scores indicated patients' better life quality.

### 2.6. Statistical Methods

SPSS21.0 software was adopted to statistically analyze and process the data in this study. GraphPad Prism 6 (GraphPad Software, San Diego, USA) was also used to draw pictures of the data. Measurement data were expressed by ($\bar{\text{x}}$ ± *s*) and tested by *t*-test. Enumeration data were expressed as [*n* (%)] and tested by *X*^2^ test. The differences had statistical significance when *P* < 0.05.

## 3. Results

### 3.1. Comparison of Clinical Data between the Two Groups

There were no significant differences in sex ratio, age, BMI, smoking history, drinking history, marital status, and disease types between the two groups (*P* > 0.05), as shown in Table 1.

### 3.2. Comparison of VAS Scores between the Two Groups before and after Intervention

**Figure 1:**
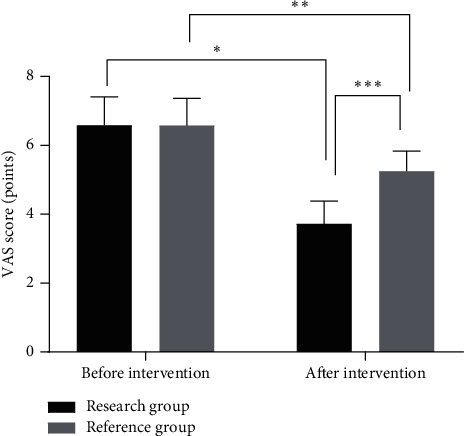
Comparison of VAS scores between the two groups before and after intervention ($\bar{x}$ ± *s*). Note: the abscissa indicates before and after intervention, while the ordinate indicates the VAS score. The VAS scores in the research group before and after intervention were (6.03 ± 1.17) points and (3.27 ± 0.94) points, while the VAS scores in the reference group before and after intervention were (6.05 ± 1.12) points and (4.86 ± 0.83) points. ^∗^indicates that there were significant differences in VAS scores in the research group before and after intervention (*t* = 13.762, *P* < 0.001). ^∗∗^indicates that there were significant differences in VAS scores in the reference group before and after intervention (*t* = 6.388, *P* < 0.001). ^∗∗∗^indicates that there were significant differences in VAS scores between the two groups before and after intervention (*t* = 9.488, *P* < 0.001).

The VAS scores after intervention in both groups were significantly lower than those before intervention (*P* < 0.05), and the VAS scores after intervention in the research group were significantly lower than those in the reference group (*P* < 0.05), as shown in Figure 1.

### 3.3. Comparison of Clinical Nursing Quality between the Two Groups

**Figure 2:**
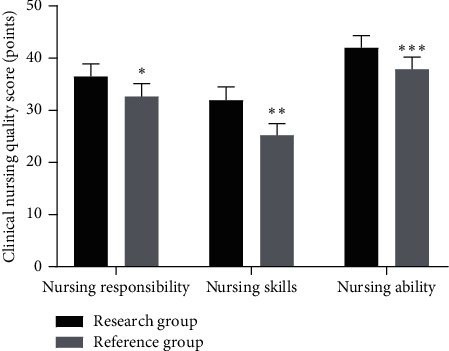
Comparison of clinical nursing quality between the two groups ($\bar{x}$ ± *s*). Note: the abscissa indicates nursing responsibility, nursing skills, and nursing ability, while the ordinate indicates clinical nursing quality score (points). The scores of nursing responsibility, nursing skills, and nursing ability in the research group were (34.94 ± 3.46), (30.28 ± 3.64), and (40.62 ± 3.25), respectively. The scores of nursing responsibility, nursing skills, and nursing ability in the reference group were (31.05 ± 3.53), (23.74 ± 3.19), and (36.51 ± 3.21), respectively. ^∗^indicates that there were significant differences in nursing responsibility between the two groups (*t* = 5.889, *P* < 0.001). ^∗∗^indicates that there were significant differences in nursing skills between the two groups (*t* = 10.112, *P* < 0.001). ^∗∗∗^indicates that there were significant differences in nursing ability between the two groups (*t* = 6.733, *P* < 0.001).

The nursing ability, nursing skills, and nursing responsibility in the research group were significantly higher than those in the reference group (*P* < 0.05), as shown in Figure 2.

### 3.4. Comparison of Clinical Nursing Satisfaction between the Two Groups

There were no significant differences between the two groups in the number of patients who were satisfied and needed improvement (*P* > 0.05). The number of very satisfied cases in the research group was significantly higher than that in the reference group (*P* < 0.05), and the number of unsatisfied cases was significantly lower than that in the reference group (*P* < 0.05), as shown in Table 2.

### 3.5. Comparison of Clinical Adverse Events between the Two Groups

The total incidence of clinical adverse events in the research group was significantly lower than that in the reference group (*P* < 0.05), as shown in Table 3.

### 3.6. Comparison of Gastrointestinal Disease Related Knowledge Scores between the Two Groups before and after Intervention

**Figure 3:**
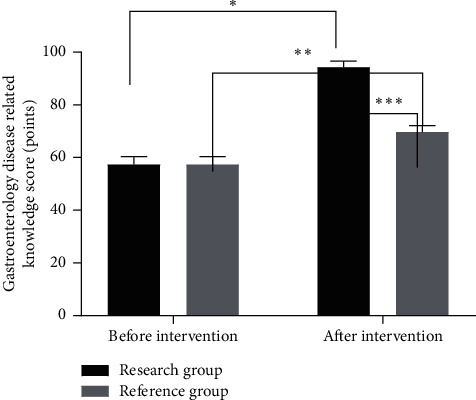
Comparison of gastrointestinal disease related knowledge scores between the two groups ($\bar{x}$ ± *s*). Note: the abscissa indicates before and after intervention, while the ordinate indicates the gastrointestinal disease related knowledge scores. The gastrointestinal disease related knowledge scores in the research group before and after intervention were (55.47 ± 4.36) points and (93.23 ± 3.17) points, respectively. The gastrointestinal disease related knowledge scores in the reference group before and after intervention were (55.51 ± 4.23) points and (68.35 ± 3.43) points, respectively. ^∗^indicates that there were significant differences in the gastrointestinal disease related knowledge scores in the research group before and after intervention (*t* = 52.419, *P* < 0.01). ^∗∗^indicates that there were significant differences in the gastrointestinal disease related knowledge scores in the reference group before and after intervention (*t* = 17.419, *P* < 0.001). ^∗∗∗^indicates that there were significant differences in the gastrointestinal disease related knowledge scores between the two groups after intervention (*t* = 39.864, *P* < 0.001).

The gastrointestinal disease related knowledge scores in the two groups after intervention were significantly higher than those before intervention (*P* < 0.05), and the gastrointestinal disease related knowledge scores in the research group after intervention were significantly higher than those in the reference group (*P* < 0.05), as shown in Figure 3.

### 3.7. Comparison of Life Quality Scores between the Two Groups before and after Intervention

**Figure 4:**
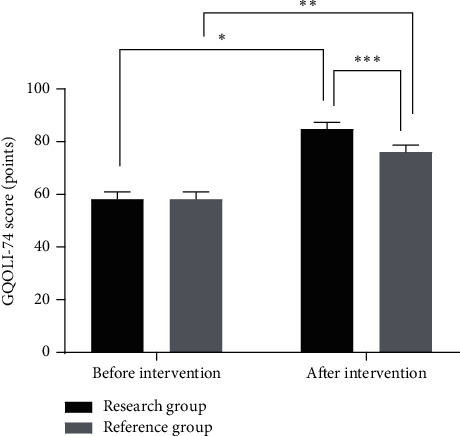
Comparison of life quality scores between the two groups before and after intervention ($\bar{x}$ ± *s*). Note: the abscissa indicates before and after intervention, while the ordinate indicates GQOLI-74 score. The GQOLI-74 scores in the research group before and after intervention were (56.54 ± 3.86) points and (83.47 ± 3.55) points, respectively. The GQOLI-74 scores in the reference group before and after intervention were (56.51 ± 3.90) points and (74.53 ± 3.84) points, respectively. ^∗^indicates that there were significant differences in GQOLI-74 scores in the research group before and after intervention (*t* = 38.428, *P* < 0.01). ^*∗∗*^indicates that there were significant differences in the GQOLI-74 scores in the reference group before and after intervention (*t* = 24.638, *P* < 0.01). ^∗∗∗^indicates that there were significant differences in GQOLI-74 scores between the two groups after intervention (*t* = 12.793, *P* < 0.01).

The GQOLI-74 scores in the two groups after intervention were significantly higher than those before intervention (*P* < 0.05), and the GQOLI-74 scores in the research group after intervention were significantly higher than those in the reference group (*P* < 0.05), as shown in Figure 4.

## 4. Discussion

The gastroenterology department is one of the important departments in the hospital which can treat a wide range of diseases, with a high recurrence rate. Therefore, while treating gastrointestinal diseases, patients should be given scientific and precise clinical nursing care [11–14]. Detail nursing, as a patient-centered nursing concept and a nursing standard, requires nurses to do their utmost to nurse patients and make patients feel more care from hospitals, which can improve the clinical treatment effect and gain acceptance from patients and their families on hospital nursing work, thus promoting the improvement of hospital nursing service quality [15, 16]. If the patients with digestive system diseases are in critical conditions, there might be so many risks in clinical nursing management; therefore, it is particularly important to do a good job in basic nursing. Traditional clinical nursing just simply divides nurses' work into different types, which results in confusion of responsibilities, difficulty in adapting to the needs of clinical nursing management, and irritation of nurse-patient conflicts, adversely affecting treatment [17]. In bedside nursing, primary nursing teams are established and nurses are assigned with different tasks according to their working ability and patients' needs so that each patient is nursed by a primary nurse. In addition, the implementation of three-level quality control and supervision system can effectively improve the working ability of the nursing staff, and reduce or avoid the occurrence of clinical adverse events, thereby improving the nursing quality and ensuring the clinical treatment effect [18, 19]. In this study, after the implementation of combined nursing intervention for patients with gastrointestinal diseases, the VAS scores in the research group were significantly lower than those in the reference group. Pains would lead to adverse emotions which were partly negative to the prognosis of the patients. Moreover, the results of GQOLI-74 scores showed that the patients who received combined nursing intervention express satisfactory prognosis. The combined nursing intervention can relieve the negative moods by communicating with the patients and distracting patients' attention to alleviate the pains.

In addition, bedside nursing and optimization of hierarchical nursing management should also be implemented. Primary nurses should not only take charge of patient care and treatment but also promptly carry out self-examination and self-correction. The team leaders need to supervise the work of primary nurses to correct deficiencies. For the department leaders, they should supervise the daily work of primary nursing, conduct a check-up system to comprehensively grasp the causes of clinical adverse events, analyze the risk factors affecting the quality of care, and fundamentally ensure the smooth development of nursing services [18, 20, 21]. This study showed that the incidence of clinical adverse events in the research group was significantly lower than that in the reference group, suggesting that the combined nursing intervention can effectively improve the responsibility consciousness of nursing staff and reduce the incidence of clinical adverse events. This study also revealed that the combined nursing mode can significantly improve patients' clinical nursing satisfaction. Maddock et al. [22] believed that digestive system diseases, due to the long disease course, repeated medication, and high recurrence rate, easily led to patients' loss of confidence in treatment and fear of their own diseases. With the application of bedside nursing in patients with acute pancreatitis, it was found that the number of patients who were very satisfied with this nursing mode was 36, which was significantly higher than 20 in the control group, and the number of patients who were unsatisfied was 4, which was significantly lower than 18 in the control group, showing that the bedside nursing could promote the establishment of a good nurse-patient relationship and improve the patients' nursing satisfaction. In addition, the combined nursing intervention can also improve the patients' understanding of their own diseases, improve their life quality, and increase the life happiness index to a certain extent [23].

In conclusion, bedside nursing combined with detail nursing can effectively reduce patients' pains of gastrointestinal diseases, improve nursing quality and nursing satisfaction, and reduce the incidence of clinical adverse events, which is worthy of popularization and application. This study investigated the effect of bedside nursing combined with detail nursing in clinical nursing of the gastroenterology department and provide some reference for nursing improvement.

## Figures and Tables

**Table 1:** Comparison of clinical data between the two groups [*n* (%), ($\bar{\text{x}}$ ± *s*)].

Types	*n*	Research group (*n* = 56)	Reference group (*n* = 56)	*χ* ^2^/*t*	*P*
Gender				0.146	0.703
Male		31 (55.36%)	33 (58.93%)		
Female		25 (44.64%)	23 (41.07%)		

Average age (years old)		40.73 ± 4.31	40.77 ± 4.35	0.049	0.961

BMI (kg/m^2^)		22.42 ± 1.65	22.46 ± 1.63	0.129	0.898

Smoking history				0.148	0.701
No		34 (60.71%)	32 (57.14%)		
Yes		22 (39.29%)	24 (42.86%)		

Drinking history				0.156	0.693
No		37 (66.07%)	35 (62.50%)		
Yes		19 (33.93%)	21 (37.50%)		

Marital status				0.373	0.541
Unmarried		49 (87.50%)	51 (91.07%)		
Married		7 (12.50%)	5 (8.93%)		

Disease types					
Gastric polyps		16 (28.57%)	18 (32.14%)	0.169	0.681
Duodenal ulcer		19 (33.93%)	16 (28.57%)	0.374	0.541
Gastric ulcer		14 (25.00%)	17 (30.36%)	0.401	0.526
Esophagitis		7 (12.50%)	5 (8.93%)	0.373	0.541

**Table 2:** Comparison of clinical nursing satisfaction between the two groups [*n* (%)].

Satisfaction	Research group (*n* = 56)	Reference group (*n* = 56)	*χ* ^**2**^	*P*
Very satisfied	33 (58.93%)	18 (32.14%)	8.1000	0.004
Satisfied	13 (23.21%)	15 (26.79%)	0.191	0.663
Needing improvement	7 (12.50%)	9 (16.07%)	0.292	0.589
Unsatisfied	3 (5.36%)	14 (25.00%)	8.391	0.004

**Table 3:** Comparison of clinical adverse events between the two groups [*n* (%)].

Group	*n*	Medication errors	Aspiration	Empyrosis	Falling down	Total incidence
Research group	56	0 (0.00%)	1 (1.79%)	0 (0.00%)	1 (1.79%)	3.57% (2/56)
Reference group	56	2 (3.57%)	3 (5.36%)	2 (3.57%)	1 (1.79%)	14.29% (8/56)
*χ* ^2^						3.953
*P*						0.047

## Data Availability

The primary data to support the results of this study are available at reasonable request to the corresponding author.
